# The Effect of Consuming Caffeine Before Late Afternoon/Evening Training or Competition on Sleep: A Systematic Review with Meta-Analysis

**DOI:** 10.3390/sports13090317

**Published:** 2025-09-10

**Authors:** Adem Kocak, Ekavi Georgousopoulou, Catherine R. Knight-Agarwal, Raymond Matthews, Michelle Minehan

**Affiliations:** 1 Nutrition & Dietetics, Faculty of Health, University of Canberra, Canberra, ACT 2617, Australia; ekavi.georgousopoulou@canberra.edu.au (E.G.); cathy.knight-agarwal@canberra.edu.au (C.R.K.-A.); 2 Appleton Institute, Central Queensland University, Rockhampton, QLD 4702, Australia; raymond.matthews2@defence.gov.au; 3 Research Institute for Sport and Exercise, University of Canberra, Canberra, ACT 2617, Australia; michelle.minehan@canberra.edu.au

**Keywords:** caffeine, sleep quality, ergogenic aid, athlete recovery, actigraphy, polysomnography, sleep disruption

## Abstract

Many athletes consume caffeine before late afternoon/evening training sessions or competition, yet the impact on subsequent sleep remains unclear. This systematic review with meta-analysis examined the effects of late afternoon/evening caffeine consumption on sleep in athletes. Ten studies (*n* = 128 athletes) involving randomized controlled trials and quasi-experimental designs were included if caffeine was consumed prior to late afternoon/evening training and subsequent sleep was measured. Meta-analysis followed PRISMA guidelines with risk of bias assessed using RoB-2 and ROBINS-I tools. Meta-analysis of randomized controlled trials revealed a small reduction in sleep efficiency with evening caffeine consumption: mean difference −4.87%, 95% CI −7.45 to −2.29, *p* = 0.04, though this effect was not robust in leave-one-out sensitivity analyses. Total sleep time showed a non-significant trend toward reduction: mean difference −32.47 min, 95% CI −69.93 to 4.99, *p* = 0.08, I^2^ = 0%. Athletes consistently reported substantial subjective sleep impairment following evening caffeine intake (3–6 mg/kg BM), creating a pronounced objective–subjective disconnect. The most notable finding is that athletes consistently perceive substantial sleep disruption despite inconsistent objective changes, highlighting the importance of subjective sleep experience in athletic populations. These findings should be interpreted cautiously given the small number of studies and predominance of male participants, limiting generalisability.

## 1. Introduction

Sleep is a fundamental biological process essential for physiological homeostasis, cognitive function, and overall health [1]. While general population sleep guidelines recommend seven to nine hours nightly, athletes require both sufficient duration and quality sleep for optimal recovery and performance [2]. However, athletes frequently fail to meet sleep recommendations, with studies consistently demonstrating inadequate sleep duration and poor sleep quality across various sporting disciplines [3]. This concern is amplified by the understanding that caffeine is an effective ergogenic aid for athletes, yet with potential to impair sleep [4].

Sleep assessment in athletes utilizes both objective measures (polysomnography, actigraphy) and subjective tools (questionnaires, sleep diaries), with key parameters including total sleep time, sleep efficiency, and sleep onset latency [5]. The complementary use of both approaches is essential given that objective and subjective sleep measures can diverge, particularly following interventions like caffeine consumption. This methodological consideration becomes particularly relevant when evaluating the practical implications of sleep disruption in athletic contexts, where athlete perception of sleep quality may be as important as objective measurements [6,7].

Caffeine is widely recognized as one of the most effective ergogenic aids available to athletes, with extensive research demonstrating performance benefits across endurance, strength, power, and cognitive-motor domains [8]. Caffeine is typically effective at doses of 3–6 mg/kg body mass, reaching peak plasma concentrations 60–120 min post-ingestion with a half-life of 4–6 h [9]. The primary mechanism involves adenosine receptor antagonism in the central nervous system, preventing the accumulation of sleep-promoting adenosine and maintaining alertness [8]. Caffeine is not prohibited by WADA; since 2004 it has been off the Prohibited List and appears only in the Monitoring Program. [10].

Athletes seeking to use caffeine to support late afternoon/evening training sessions need to consider if the performance benefit warrants the risk of disrupting subsequent sleep [11]. In practice, there exists tension between athletes and coaches, with coaches often discouraging evening caffeine use while athletes may ignore this advice to maximize training benefits. This conflict represents a common real-world scenario where evidence-based guidance is needed to inform decision-making.

A critical gap in understanding caffeine’s impact on athletic sleep concerns the physiological interaction between two sleep stressors: late-evening high-intensity exercise and caffeine consumption. Evening training or competition creates sympathetic arousal, elevated core body temperature, and heightened psychological stress, all factors that independently disrupt sleep architecture [12]. When combined with caffeine’s adenosine receptor antagonism, these dual stressors may create a compounded effect on sleep disruption that extends beyond what either factor produces in isolation. Research demonstrates that competition environments amplify caffeine’s sleep-disrupting effects beyond laboratory conditions [13]. Post-competition cortisol levels remained elevated 3–4 h longer than post-training levels, creating an additive stress response that compounds caffeine’s adenosine receptor antagonism [14]. Additionally, the potential disconnect between objective measurements and subjective sleep experiences may be particularly pronounced in athletic populations, where athletes’ perceptions of recovery and readiness are closely linked to their sleep experience [3,15]. Given that athletes’ subjective assessment of sleep quality strongly influences their training responses and recovery behaviours, understanding both the physiological interactions and the objective–subjective relationship is crucial for evidence-based recommendations in athletic populations [16].

While systematic reviews have examined caffeine’s sleep effects in general populations [17] and broader dietary impacts on athletic sleep [18], no previous meta-analysis has specifically quantified the effects of evening caffeine consumption in athletic populations. Existing athlete-focused reviews have been largely narrative, noting trends toward sleep impairment but lacking the statistical synthesis necessary to determine consistent patterns across studies [19]. A comprehensive systematic review of dietary factors affecting athletically trained populations identified that all eight studies examining caffeine found negative effects on sleep duration and/or quality, with evening caffeine intake (≥5 p.m.) at doses >2 mg·kg^−1^ decreasing sleep duration and efficiency while increasing sleep latency [18]. However, methodological inconsistencies in sleep assessment approaches—with studies using polysomnography, actigraphy, and subjective measures showing divergent sensitivity to caffeine’s effects, have complicated interpretation of individual study findings [19].

Therefore, we aimed to systematically review studies that measured the effect of caffeine consumed prior to late afternoon/evening (>16:00 hrs) training or competition on subsequent sleep in athletes. Meta-analysis was conducted to derive pooled effect estimates and quantify the direction and magnitude of caffeine’s impact on key sleep outcomes, with particular attention to both objective sleep parameters and subjective sleep experiences. By narrowing our scope to athletic populations and late-day ingestion, representing the first systematic investigation, we aimed to improve the practical relevance of findings and contribute to evidence-based recommendations for sport practitioners and athletes.

## 2. Materials and Methods

**Figure 1:**
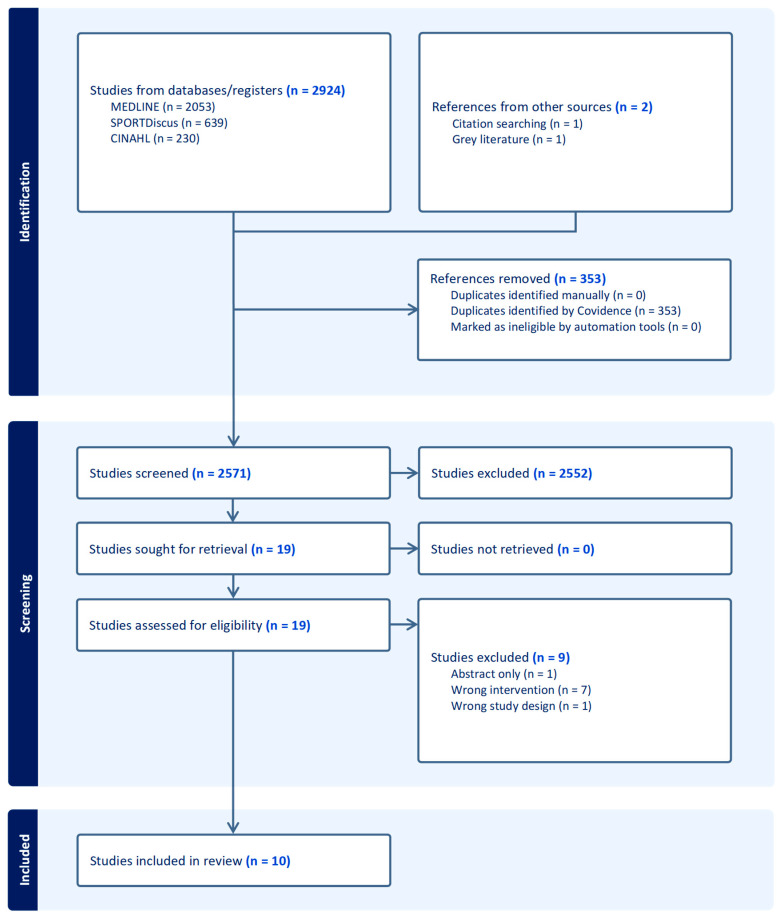
PRISMA flow chart of the search and screening process.

This review was planned, conducted and reported in accordance with the Preferred Reporting Items for Systematic Reviews and Meta-Analyses (PRISMA) statement (Figure 1: PRISMA flow diagram) [20]. It was registered with the Prospective Register for Systematic Reviews (PROSPERO) (CRD420250569960). As this study is a systematic review and meta-analysis of previously published research, ethical approval was not necessary.

### 2.1. Study Selection Criteria

Studies were included if they were randomized controlled trials (RCTs), quasi-experimental trials or observational trials with a control group. Cross-sectional, qualitative studies, and observational studies with no control group were excluded. Participants were male and/or female athletes from any sport who consumed caffeine prior to training or competition scheduled after 16:00. The dose and timing of caffeine consumption was required to be reported, or a biomarker of caffeine consumption measured (e.g., saliva caffeine). The outcomes of interest were measures of sleep quantity and quality measured by polysomnography, actigraphy or sleep questionnaire.

### 2.2. Search Strategy

A search strategy was developed with the assistance of an academic librarian. The search strategy included terms related to caffeine and other stimulants, athlete, sleep and sleep quality. Boolean operators and wild-card options were utilised to connect various keywords, and MeSH terms were incorporated where appropriate. Search terms are provided in the supplementary material (Table S1). Medline, SPORTDiscus, and CINAHL were searched for English language publications from inception to 30 September 2024. While the formal search concluded at this date, a surveillance of literature through to April 2025 did not identify any additional studies that would meet inclusion criteria. The search was limited to human studies. All search results were exported to Covidence™ (Veritas Health Innovation, Melbourne, Australia) for removal of duplicates and screening.

Two authors independently screened titles and abstracts, then full text papers according to inclusion and exclusion criteria. No conflicts occurred during the screening process. Reference lists of included papers were checked for additional papers, and two additional papers were identified.

### 2.3. Data Extraction and Quality Assessment 

Randomized controlled trials were assessed for risk of bias using the Risk of Bias in Randomised trials (RoB-2, Version 2.0, August 2019) tool [21]. Non-randomized studies were assessed using the Risk of Bias in Non-Randomized Studies—of Interventions (ROBINS-I, Version 2, November 2024) tool [22]. RoB-2 evaluates six domains of bias: (1) risk of bias arising from the randomization process; (2) risk of bias due to period and carryover effects; (3) risk of bias due to deviations from intended interventions; (4) risk of bias due to missing outcome data; (5) risk of bias arising from measurement of the outcome; and (6) risk of bias in selection of the reported result. ROBINS-I evaluates seven domains of bias: (1) risk of bias due to confounding; (2) risk of bias in classification of interventions; (3) risk of bias in selection of participants into the study (or into the analysis); (4) risk of bias due to deviations from intended interventions; (5) risk of bias due to missing data; (6) risk of bias arising from measurement of the outcome; and (7) risk of bias in selection of the reported result. Two authors independently assessed papers, and a third author moderated any discrepancies. Studies were not excluded based on their risk of bias score.

Data extraction was completed in Covidence™ by one author and checked by a second author. Extracted data included author, study design, sport, number of participants, intervention, control group, sleep measures and main findings (Table 1).

Where there was sufficient homogeneity in study design, meta-analysis was conducted. Statistical analyses were performed by one author and supported by another author using Review Manager software (RevMan V.5.4). Primary data extraction focused on mean changes in sleep parameters, with mean final values utilised when change data were unavailable, following Cochrane guidelines [34]. Additional data were solicited from study authors when necessary. All temporal measurements were standardised to minutes. Standard deviations were calculated from standard errors or confidence intervals using established Cochrane methodologies [35]. To reduce the likelihood of spurious findings from multiple testing, we restricted analyses to two primary sleep measures, following recommendations for hypothesis-driven research [36].

Forest plots were generated for individual sleep parameters. Between-study heterogeneity was quantified using the I^2^ statistic, with thresholds defined as ≥75% indicating high heterogeneity, 50–75% substantial, 36–60% moderate, and 0–35% low heterogeneity, following Higgins et al. [37]. Given the small number of trials and anticipated between-study variance, we used random-effects meta-analysis with the Hartung–Knapp–Sidik–Jonkman adjustment. Abstract *p*-values reflect this approach.

Due to the small number of studies included in the meta-analysis, formal subgroup analyses (e.g., by caffeine dose or timing) were not feasible. Sequential sensitivity analyses were conducted by systematically excluding individual studies to assess their contribution to the overall effect size (Table S2A,B).

Heterogeneity was quantified using I^2^ statistics, with values >50% considered substantial. Given the small number of studies, we used the Hartung-Knapp-Sidik-Jonkman method for random-effects meta-analysis to reduce false-positive rates. Publication bias was assessed via funnel plots, though interpretive power was limited by the inclusion of fewer than 10 studies.

## 3. Results

A total of 2571 articles were identified after removal of duplicates (Figure 1). After eligibility assessment, 10 articles were included in the final review (Table 1). Specifically, 2552 studies were excluded during title and abstract screening and 9 studies excluded during full-text assessment (wrong population *n* = 1, wrong intervention *n* = 7, wrong study design *n* = 1) [20].

Eight studies were randomized controlled trials (RCTs) with crossover conditions (six double-blind, two single-blind), while two studies utilized quasi-experimental designs. Studies were conducted in Australia (*n* = 4), Poland (*n* = 1), Spain (*n* = 2), Portugal (*n* = 1), New Zealand (*n* = 1), United Kingdom (*n* = 1). The sample sizes ranged from 8–20 participants, with a mean age range of 18–28 years. The study sample comprised athletes from combat sports, endurance sports, and team sports, with classifications ranging from tier 2 (trained) to tier 4 (elite) [33].

Caffeine doses of 3–6 mg/kg body mass were administered between 16:00–19:45 prior to training (*n* = 8) or competition (*n* = 2). All RCTs utilized placebo comparators, while the quasi-experimental studies examined high versus low post-match salivary caffeine concentrations. Sleep assessment methodologies included objective measures such as actigraphy (*n* = 5) and polysomnography (*n* = 1), as well as subjective instruments including the Karolinska Sleep Questionnaire [3] (*n* = 2), Leeds Sleep Evaluation Questionnaire [4] (*n* = 1), Side Effects Questionnaire [5] (*n* = 2), and sleep diaries (*n* = 2).

Risk of Bias Assessment

**Figure 2:**
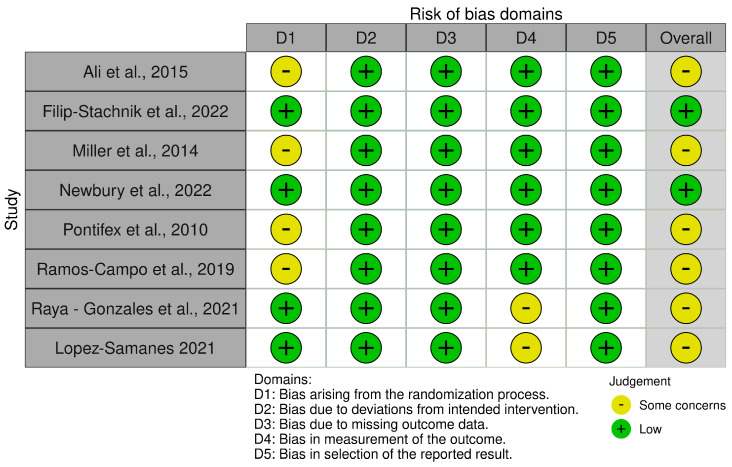
Risk of bias summary of randomized controlled trials examining the effect of evening caffeine consumption on objective and subjective sleep measures in athletic populations. References included in the figure [23,24,25,26,27,28,29,30].

**Figure 3:**
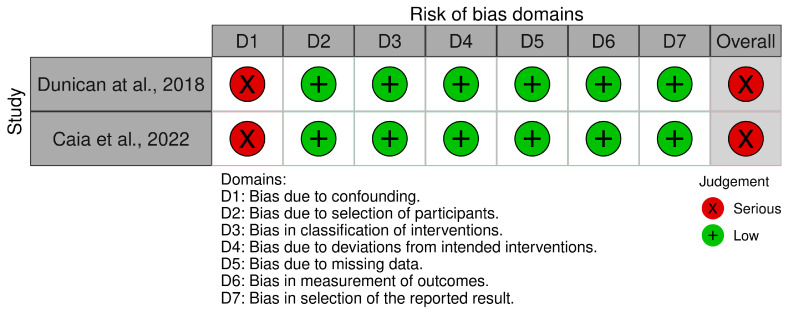
Risk of bias summary of quasi-experimental studies examining the relationship between post-match salivary caffeine levels and sleep outcomes in professional rugby athletes. References included in the figure [31,32].

When applying the RoB-2 tool, four studies demonstrated ‘low’ risk of bias across all domains, while four studies were assessed as having ‘some concerns’ due to issues primarily related to the randomization process and the measurement of the outcome (Figure 2) [21]. Papers were limited by randomization processes and reliance on questionnaires to measure sleep. The two non-randomized studies evaluated using ROBINS-I exhibited ‘serious’ risk of bias in Domain 1 (confounding factors), while maintaining ‘low’ risk across all other domains (Figure 3) [22]. The studies allowed ad libitum intake of caffeine under real-competition conditions but used salivary caffeine as an indicator of caffeine exposure. The dose and timing of caffeine consumption was unclear.

### 3.1. Total Sleep Time

**Figure 4:**
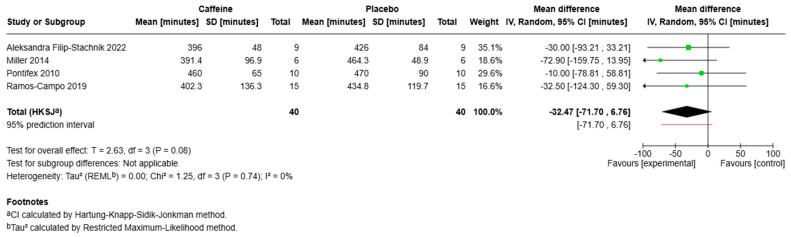
Forest plot meta-analysis of total sleep time (TST). Each green square represents a study’s effect estimate, with square size proportional to its weight; horizontal lines show the 95% CI. The vertical line at 0 min marks no effect. The black diamond indicates the pooled random-effects (IV) estimate, with width showing the 95% CI; a 95% prediction interval is shown beneath. The overall effect was not statistically significant (MD = −32.5 min, 95% CI −71.7 to 6.8; P = 0.08), and heterogeneity was low (I^2^ = 0%). References included in the figure [23,24,25,26].

Total Sleep Time (TST) was assessed in six studies via actigraphy (*n* = 5), polysomnography (*n* = 1), and sleep diary (*n* = 1). The three RCTs and two quasi-experimental studies that measured TST found no significant differences in actigraphy-measured TST between caffeine and placebo conditions, whereas the polysomnography-based study reported a significant reduction in sleep duration. Meta-analysis of the effect of caffeine on TST was completed on four RCTs with comparable study design. These studies included 36 male and 4 female participants, with a mean age range of 18 to 28 years. The meta-analysis indicated a trend for a reduction in TST following caffeine ingestion (mean difference: −32.47 min, 95% CI: −69.93 to 4.99, *p* = 0.08) (Figure 4). However, the reduction did not reach statistical significance. Notably, heterogeneity across studies was low (χ^2^ = 1.25, df = 3, *p* = 0.74, I^2^ = 0%), indicating consistency in the observed effects despite variations in caffeine dose and timing of ingestion. Sensitivity analyses showed that the direction of effect remained negative across all iterations. Statistical significance (*p* < 0.05) was not retained when any single study was removed (Table S2A) [38].

### 3.2. Sleep Efficiency

**Figure 5:**
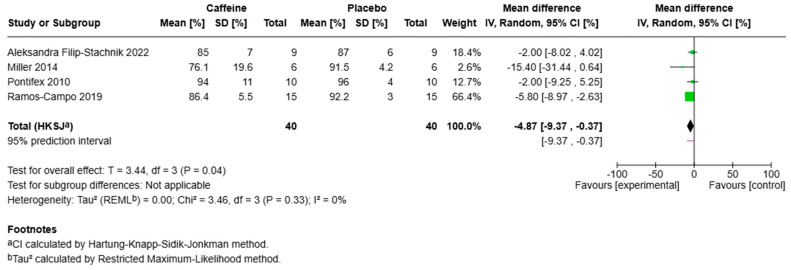
Forest plot meta-analysis of sleep efficiency (SE). Each green square represents a study’s effect estimate, with square size proportional to its weight; horizontal lines show the 95% CI. The vertical line at 0% indicates no effect. The black diamond shows the pooled random-effects (IV) estimate, with width indicating the 95% CI; a 95% prediction interval is displayed beneath if present. The overall effect was statistically significant (*p* < 0.05) with low heterogeneity (I^2^ = 13%). References included in the figure [23,24,25,26].

Seven studies assessed Sleep Efficiency (SE) with mixed results. Two RCTs and one quasi-experimental design reported significant reductions in SE following caffeine exposure. The remaining studies reported no effect on SE. Results from four RCTs were included in a meta-analysis of caffeine and SE. This indicated a potential reduction in SE following caffeine ingestion compared to placebo (mean difference: −4.87%, 95% CI: −7.45 to −2.29, *p* = 0.04) (Figure 5). Heterogeneity among included studies was low (χ^2^ = 3.46, df = 3, *p* = 0.33, I^2^ = 13%). However, sensitivity analyses revealed that statistical significance (*p* < 0.05) did not remain when individual studies were sequentially excluded (Table S2B), indicating that this finding lacks robustness and is disproportionately influenced by specific studies rather than reflecting a consistent effect across the literature [39].

### 3.3. Wake After Sleep Onset

Seven studies objectively measured Wake After Sleep Onset (WASO). Two RCTs reported significant increases in WASO following caffeine ingestion—one using polysomnography and one using actigraphy. In contrast, five studies, including three RCTs using actigraphy and two quasi-experimental designs, found no significant differences in WASO between caffeine and placebo conditions.

### 3.4. Sleep Onset Latency

Four studies assessed Sleep Onset Latency (SOL). One RCT found a significant increase in SOL following caffeine ingestion, whereas two others reported no significant changes using actigraphy and subjective sleep measures. The quasi-experimental studies observed opposing effects on SOL.

### 3.5. Number of Awakenings

Four studies examined the number of wake times. Only one study, using actigraphy, reported a significant increase in the number of awakenings following caffeine ingestion. The remaining three studies, two using actigraphy and one using polysomnography, found no significant difference between conditions.

### 3.6. Subjective Sleep

Six studies assessed subjective sleep outcomes using self-reported measures. Four studies using the Karolinska Sleep Questionnaire (KSQ), Leeds Sleep Evaluation Questionnaire, and Side Effects Questionnaire found reductions in parameters such as sleep quality, sleep latency, and time to fall asleep. However, no significant changes in perceived sleep quality were reported in the remaining two studies.

The overall certainty of evidence for the impact of evening caffeine intake on total sleep time and sleep efficiency, appraised with GRADE, was low to very low owing to small sample sizes, imprecision, and risk of bias in non-randomized evidence. This rating reflects concerns with risk of bias, inconsistency of findings, and imprecision due to small sample sizes. Accordingly, clinical recommendations should remain cautious until larger, well-controlled trials are available [40].

## 4. Discussion

This systematic review with meta-analysis provides important insight into the complex relationship between late afternoon/evening caffeine consumption and sleep in athletes. The most robust and clinically meaningful finding is the pronounced disconnect between objective sleep measurements and athletes’ subjective sleep experiences following evening caffeine consumption—a finding that emerged consistently across multiple studies and represents a more reliable result than the fragile objective sleep efficiency reduction identified in meta-analysis.

### 4.1. Subjective–Objective Disconnect

The most consistent and practically significant finding of our systematic review is the robust disconnect between objective sleep measurements and athletes’ subjective sleep experiences following evening caffeine consumption. This disconnect represents more than a methodological curiosity—it constitutes the most clinically relevant finding for athletic populations and challenges the primacy of objective sleep assessment in sports contexts.

Several studies exemplify this phenomenon. Filip-Stachnik et al. (2022) reported no significant differences in actigraphy-derived sleep parameters following evening caffeine intake, yet participants reported significantly poorer sleep quality on the Karolinska Sleep Questionnaire (*p* = 0.03) [23]. Similarly, Ramos-Campo et al. (2019) found modest objective changes—reduced sleep efficiency (92.2% to 86.4%, *p* = 0.003), increased wake time (29.2 to 52.1 min, *p* = 0.001), yet subjective measures revealed substantially greater impairments, with large effect sizes for sleep quality, calmness, ease of falling asleep, and refreshment upon waking [26]. In contrast, Pontifex et al. (2010), which relied solely on actigraphy, detected no significant changes, highlighting the risk of underestimating perceptual sleep disruption when using objective measures alone [25].

The clinical significance of this disconnect cannot be overstated. For athletes, perceived sleep quality strongly influences recovery behaviours, training readiness, and performance expectations [7]. An athlete who reports feeling unrested and experiencing poor sleep quality—regardless of objective sleep parameters, may exhibit reduced training motivation, altered nutrition choices, and compromised psychological readiness. These subjective experiences represent real, actionable concerns that warrant equal consideration alongside objective findings.

This objective–subjective discord may be particularly pronounced in athletic populations due to heightened interoceptive awareness, where athletes’ enhanced ability to detect physiological changes makes them more sensitive to caffeine’s subtle stimulatory effects that may not register on actigraphy or basic polysomnography measures [6]. The consistency of subjective complaints across multiple studies, even when objective effects were minimal, reinforces the clinical relevance of athlete-reported sleep experiences and challenges the practice of dismissing subjective reports when objective measures appear unchanged.

### 4.2. Sleep Outcomes and Meta-Analysis Findings

The pooled analysis of Total Sleep Time (TST) revealed a non-significant reduction associated with evening caffeine consumption, which may offer some reassurance to athletes seeking performance benefits from late-day caffeine use. However, the observed trend toward a mean decrease of approximately 32 min (95% CI: −69.93 to 4.99, *p* = 0.08) could have practical relevance, particularly if such reductions accumulate over multiple days of training and competition [38]. Variability in sleep assessment methods likely influenced these findings. Notably, the sole polysomnography study reported a larger TST reduction compared to studies relying on actigraphy, consistent with known limitations of actigraphy to detect wakefulness during immobile periods [24,41]. This methodological difference underscores the challenge of accurately quantifying sleep disruption in real-world athletic settings.

Regarding sleep efficiency, our meta-analysis suggests a potential reduction in SE following caffeine consumption before late afternoon/evening sessions. While this finding aligns with caffeine’s known stimulant effects, including prolonged sleep onset latency and increased nocturnal wakefulness, sensitivity analyses revealed that statistical significance was not maintained when individual studies were removed sequentially, indicating that this result lacks robustness and should be interpreted with considerable caution [39]. The fragility of this finding suggests it is disproportionately influenced by specific studies rather than reflecting a consistent and stable effect across the broader literature.

While our meta-analyses revealed low statistical heterogeneity for both total sleep time (I^2^ = 0%) and sleep efficiency (I^2^ = 13%), considerable clinical heterogeneity existed across the included studies. This apparent discrepancy warrants careful interpretation, as the low I^2^ values may reflect the limited number of studies rather than true homogeneity of effects [42]. Clinical heterogeneity was evident across multiple dimensions. Caffeine doses varied two-fold (3 vs. 6 mg/kg), sport modalities ranged from individual endurance activities to team sports with different physiological demands, and sleep assessment methods included polysomnography, actigraphy, and various subjective questionnaires. Additionally, timing of caffeine consumption varied from 16:00 to 19:45, potentially influencing the duration between ingestion and typical bedtime.

The I^2^ statistic has limited power to detect heterogeneity when few studies are included, and may underestimate true between-study variance in small meta-analyses [39]. These clinical differences likely contributed to the variability in individual study outcomes, even though statistical tests failed to capture this heterogeneity. For instance, the single polysomnography study reported larger sleep disruptions compared to actigraphy-based studies, while the magnitude of subjective sleep impairment varied considerably despite similar objective findings.

### 4.3. Neurophysiological Mechanisms

Caffeine’s primary mechanism of sleep disruption operates through competitive adenosine receptor antagonism in the central nervous system, specifically targeting A1 and A2A receptors [43]. Under normal physiological conditions, adenosine accumulates during wakefulness as a metabolic byproduct, with A1 receptors predominantly located in the basal forebrain and cortex inhibiting wake-active cholinergic neurons, while A2A receptors concentrated in the ventrolateral preoptic area (VLPO) activate GABAergic sleep-promoting neurons [11].

Controlled sleep deprivation studies demonstrate that caffeine’s adenosine receptor blockade specifically reduces slow-wave activity and increases spindle frequency activity in non-REM sleep, indicating that caffeine attenuates sleep depth and restorative quality even when plasma concentrations approach undetectable levels [43]. This explains why caffeine consumed hours before bedtime can still disrupt sleep architecture through interference with homeostatic sleep drive mechanisms, supporting our meta-analytic finding of reduced sleep efficiency despite variable timing across studies.

Beyond adenosine receptor antagonism, caffeine exerts effects through additional neurophysiological pathways. Methylxanthine compounds promote intracellular calcium mobilization through the sarcoplasmic reticulum, facilitating controlled neurotransmitter release via synaptic transmission [44]. Additionally, phosphodiesterase inhibition prevents cyclic adenosine monophosphate (cAMP) breakdown, stimulating dopamine, epinephrine, and norepinephrine release [44]. However, these mechanisms require concentrations approaching toxic levels, making adenosine receptor antagonism the primary clinically relevant pathway.

The adenosine receptor blockade creates a complex neurotransmitter cascade affecting sleep architecture by indirectly influencing norepinephrine, dopamine, acetylcholine, serotonin, glutamate, and GABA release [44]. This multi-neurotransmitter effect explains why caffeine’s sleep disruption extends beyond simple arousal to affect mood, memory consolidation, and subjective sleep quality parameters consistently observed across studies in our meta-analysis.

### 4.4. Individual Variability and Metabolism

Metabolic variability, primarily mediated by CYP1A2 polymorphisms, represents a critical determinant of caffeine’s effects [45]. The pharmacokinetic impact of oral contraceptive steroids (OCS) on caffeine elimination is particularly significant, as demonstrated by Patwardhan et al.’s (1980) seminal study showing a near-doubling of caffeine’s elimination half-life in OCS users (10.7 ± 3.0 h vs. 6.2 ± 1.6 h in non-OCS females, *p* < 0.001) and a 40% reduction in total plasma clearance (0.79 vs. 1.3 mL/min/kg) [46]. Ali et al.’s (2015) study of female athletes using oral contraceptives revealed that estrogen-mediated CYP1A2 inhibition extended caffeine’s half-life to 17.63 ± 8.06 h, resulting in prolonged plasma caffeine concentrations that correlated strongly with increased sleep latency (r = 0.48) [27].

These pharmacokinetic differences may partly explain why some athletes report poor sleep despite fewer changes in objective values. Filip-Stachnik et al. postulated that subjective impairments may arise from heightened perceptual sensitivity to caffeine’s stimulatory effects, particularly in habitual users or slower metabolisers [23]. Genetic polymorphisms such as those in CYP1A2 and ADORA2A may modulate these sensitivity responses, with slow metabolisers displaying prolonged caffeine half-lives and exacerbated cardiovascular responses. Variants in ADORA2A, particularly the TT genotype, have been linked to an increased risk of sleep disruption regardless of habitual caffeine intake [47].

### 4.5. Real-World Competition Context

Competition environments amplify caffeine’s sleep-disrupting effects beyond laboratory settings through the interaction of physiological and psychological stressors [18]. Research demonstrates that competition environments amplify caffeine’s sleep-disrupting effects beyond laboratory conditions, with post-competition cortisol levels remaining elevated 3–4 h longer than post-training levels, creating an additive stress response that compounds caffeine’s adenosine receptor antagonism [15].

Two studies in this review examined real-world caffeine use during evening competition, revealing substantial individual variability in both caffeine concentrations (2.77–8.1 μg/mL) and sleep responses [31,32]. While Dunican et al. found significant correlations between post-match caffeine levels and impaired sleep (r = 0.53 for sleep latency), Caia et al. reported no such associations, highlighting the unpredictable nature of caffeine effects in competitive contexts.

Marked inter-individual variability was evident across all studies. In Dunican et al., 20% of players experienced total sleep deprivation post-match, while others obtained limited sleep under similar conditions. This variability aligns with broader sports literature demonstrating that athletes experience significantly greater sleep disruption on competition nights compared to training days, including delayed sleep onset (1.76–2.05 h later), reduced total sleep time (up to 1.86 h shorter), and elevated cognitive and somatic pre-sleep arousal [48]. Caffeine intake was significantly higher during competition (*p* < 0.001), with athletes consuming more caffeine on competition days as part of pre-competition routines to enhance performance or manage arousal, potentially amplifying sleep disruption alongside physiological and psychological competition stressors [3]. These findings suggest that caffeine’s impact on athletic sleep is context-dependent and highly individual, supporting the need for personalized management strategies rather than universal timing recommendations.

### 4.6. Methodological Considerations and Timing Effects

The timing of caffeine consumption relative to bedtime emerged as a critical factor across studies. Controlled dose–response studies demonstrate that caffeine consumed 6 h before bedtime reduced sleep efficiency by 2.6% and total sleep time by 41 min, while equivalent doses consumed 3 h before bedtime produced similar disruption [49]. Individual half-life variations create differences in cut-off times between fast and slow metabolisers [50].

However, methodological limitations in the included studies, particularly the lack of biological caffeine markers and high inter-individual sleep variability in athletes limit definitive timing recommendations [2]. Athletes exhibit greater sleep variability than age- and sex-matched controls, which likely extends to caffeine-related sleep outcomes where inter-and intra-individual differences in metabolism and sensitivity further amplify night-to-night unpredictability [51]. Future research should incorporate pharmacokinetic measures and account for the substantial night-to-night sleep variability characteristic of athletic populations to better inform personalized caffeine timing strategies.

### 4.7. Caffeine Interactions with Concurrent Supplements

Athletes frequently combine caffeine with other ergogenic aids, yet no studies in this review examined sleep outcomes following multi-supplement protocols. This represents a significant research gap given the prevalence of caffeine-containing pre-workout formulations in evening training contexts.

The pharmacological interaction between caffeine and commonly used supplements presents contradictory sleep mechanisms. While L-theanine demonstrates potential to improve sleep onset latency and overall sleep quality through GABA modulation [52], creatine may indirectly influence sleep by supporting brain energy metabolism and maintaining energy substrate availability [53].

Pre-workout formulations commonly contain caffeine (150–400 mg) alongside additional compounds (taurine, beta-alanine, citrulline), creating complex pharmacological profiles with unknown sleep implications [54]. Future research should investigate whether specific supplement combinations amplify caffeine’s sleep-disrupting effects or whether compounds like L-theanine may provide protective benefits while maintaining performance enhancement.

### 4.8. Practical Implications

This systematic review provides evidence-based guidance for optimizing evening caffeine use while minimizing sleep disruption in athletes, with three key findings having direct practical relevance.

Athletes should strategically manage caffeine timing before evening training when sleep quality and recovery are priorities. Controlled dose–response studies demonstrate that caffeine consumed 6 h before bedtime reduced sleep efficiency by 2.6% and total sleep time by 41 min, while individual half-life variations create differences in cut-off times between fast and slow metabolisers [49,50]. Athletes should avoid caffeine before evening training when sleep quality and recovery are priorities, recognizing that complete avoidance may not always be feasible given performance demands.

The consistent pattern where subjective measures indicated greater sleep disruption than objective measures has important practical implications. Athletes may report significant perceived sleep disruption despite minimal objective impairments in sleep parameters, highlighting the need for comprehensive assessment approaches that incorporate both objective monitoring and validated subjective instruments such as the Karolinska Sleep Questionnaire [55].

Athletes should establish personalized caffeine cut-off times through systematic testing during training periods using 3 mg/kg body mass consumed 4–6 h before typical bedtime, monitoring both objective and subjective sleep quality. Female athletes using oral contraceptives may require extended elimination windows (8–10 h) due to the prolonged half-life observed in this population [27,46]. When evening competition necessitates caffeine use, employ minimum effective dosing (3 mg/kg) with earliest possible consumption timing, recognizing that competition environments amplify caffeine’s sleep-disrupting effects.

### 4.9. Limitations and Future Directions

This review has several limitations. The small number of included studies (*n* = 10), each with modest sample sizes (8–20 participants), may reduce statistical power and obscure true effects. The predominance of male participants across studies also restricts the generalizability of findings to female athletes. Sensitivity analyses further revealed that the significant effect of caffeine on sleep efficiency was not robust, as the effect dissipated when individual studies were sequentially removed, indicating that results should be interpreted with caution.

Furthermore, most studies lacked physiological markers such as blood or salivary caffeine concentrations to verify actual caffeine exposure and clearance rates, making interpretation of sleep effects difficult. For quasi-experimental studies, risk of bias due to confounding was rated as ‘serious’, and uncertainty regarding ad libitum caffeine intake, timing relative to bedtime, and dose standardization likely reduced causal inference and may explain part of the variability across studies. Only two studies measured post-competition salivary caffeine, revealing substantial inter-individual variability that likely influences sleep outcomes but cannot be accounted for without systematic biomarker assessment. The absence of pharmacokinetic data prevents determination of whether sleep disruption correlates with residual caffeine concentrations or individual metabolic differences, limiting our understanding of metabolism and clearance in real-world contexts.

In several cases, sleep outcomes were reported as secondary findings in performance studies without validated sleep assessment tools, which constrains interpretability. Finally, the lack of repeated measures designs to capture night-to-night variability in sleep parameters represents a key methodological limitation, particularly relevant to athletic populations where sleep variability is inherently high [51].

Future research should prioritize studies with larger sample sizes and equal gender representation, incorporate biological caffeine markers to verify exposure and clearance, utilize validated sleep assessment instruments as primary outcomes, and employ repeated measures designs to account for night-to-night variability. Additionally, investigation of multi-supplement interactions, genetic polymorphism influences, and sport-specific responses would enhance the practical applicability of findings for athletic populations.

## 5. Conclusions

This systematic review with meta-analysis demonstrates that caffeine ingested before late afternoon/evening athletic activities creates a nuanced trade-off for athletes, with a potential reduction in sleep efficiency that requires cautious interpretation due to sensitivity analysis revealing lack of robustness, despite minimal impact on total sleep duration. Our analysis highlights a disconnect between objective sleep measurements and athlete-reported experiences, with participants frequently describing sleep disturbances that represent the most clinically meaningful finding. Findings indicate individual variability in response influenced by genetic, hormonal, and metabolic factors, suggesting athletes would benefit from individualized approaches that systematically weigh performance advantages against recovery implications, especially in scenarios with compressed recovery windows. However, these findings should be interpreted with caution given the small number of included studies, predominance of male participants, and fragile nature of objective sleep efficiency findings, which may limit generalizability to broader athletic populations.

## Figures and Tables

**Table 1:** Summary of Studies Investigating Caffeine Consumption Prior to Late Afternoon/Evening Training or Competition and Sleep Outcomes in Athletes.

Paper (Author, Year, Country)	Participants (Number, Age, Sex)	Sport (Type, Athlete Calibre) [2]	Study Design	Intervention (Dose and Timing of Caffeine or Biomarker)	Comparator	Scenario (Training or Competition, Time)	Sleep Measures	Effect of Caffeine vs. Comparator
Filip-Stachnik, 2022, Poland [23]	5 males Age: 24 ± 5 yrs 4 females Age: 20 ± 1 yrs	Judo Tier 3 (Highly Trained/National)	RCT Double-blind Cross-over	Caffeine 3 mg/kg BM Consumed 18:00	Placebo	Training Session 19:00	Actigraphy (Activeinsight’s GENEActiv watch) Karolinska Sleep Questionnaire (KSQ)	↔ No significant differences in actigraphy sleep measures KSQ: ↓ Sleep Quality (KSQ) 3.9 ± 0.6 vs. 3.0 ± 1.0 (*p* = 0.03)
Miller et al. 2014, Australia [24]	6 males Age: 28 ± 7 yrs	Cycling Triathlon Tier 2 (Trained)	RCT Double-blind Cross-over	Caffeine 2 × 3 mg/kg BM Consumed 16:00 and 17:40	Placebo	Training Session 17:00–19:00	Polysomnography	↑ Sleep latency 51.1 ± 34.7 vs. 10.2 ± 4.6 min (*p* = 0.028) ↓ REM sleep 62.1 ± 19.6 vs. 85.8 ± 24.7 min (*p* = 0.028) ↓ Total sleep time 391.4 ± 96.9 vs. 464.3 ± 48.9 min (*p* = 0.028) ↑ WASO 75.1 ± 86.6 vs. 31.9 ± 17.0 min (*p* = 0.046) ↓ Sleep efficiency 76.1 ± 19.6% vs. 91.5 ± 4.2% (*p* = 0.028)
Pontifex et al. 2010, Australia [25]	10 males Age: 18 ± 1 yrs	Australian Rules Football Soccer Field Hockey Recreational	RCT Single-blind Cross-over	Caffeine 6 mg/kg BM Consumed 16:00–19:00	Placebo	Repeated sprint exercise trail 17:00–20:00	Actigraphy	↔ No significant differences in actigraphy sleep measures
Ramos-Campo et al. 2019, Spain [26]	15 males Age: 24 ± 8 yrs	Middle Distance Runners Tier 3 (Highly Trained/National)	RCT Single-blind Cross-over	Caffeine 6 mg/kg BM Consumed 19:45	Placebo	800 m Running Time Trial 20:00	Actigraphy Karolinska Sleep Questionnaire (KSQ)	Actigraphy: ↓ Sleep Efficiency 86.4 ± 5.8% vs. 92.2 ± 3.9% (*p* = 0.003; ES = 0.71) ↓ Wake Time 52.1 ± 23.2 min vs. 29.2 ± 15.4 min (*p* = 0.001; ES = −1.18) ↑ No. Wake Times 18.85 ± 7.50 vs. 13.62 ± 7.05 (*p* = 0.005; ES = −0.96) KSQ: ↓ Sleep Quality 2.21 ± 0.98 vs. 3.36 ± 0.75 (*p* = 0.005; ES = 1.11) ↓ Calm Sleep 2.56 ± 1.15 vs. 3.50 ± 1.09 (*p* = 0.005; ES = 1.11) ↓ Ease of Falling Asleep 1.57 ± 0.85 vs. 3.43 ± 1.22 (*p* = 0.003; ES = 1.38) ↓ Feeling Refreshed After Awakening 1.50 ± 0.65 vs. 2.07 ± 0.73 (*p* = 0.006; ES = 1.11)
Ali et al. 2015, New Zealand [27]	10 females Age: 24 ± 4 yrs	Soccer Hockey Netball Recreational to International	RCT Double-blind Cross-over	Caffeine 6 mg/kg BM Consumed 17:15	Placebo	Intermittent exercise protocol to simulate soccer match 18:00	Leeds Sleep Evaluation Questionnaire	LSEQ: ↑ Sleep Latency 5.9 ± 3.2 cm vs. 3.1 ± 1.7 cm (*p* < 0.05) ↑ Time to Get to Sleep 5.9 ± 3.2 cm vs. 2.8 ± 1.5 cm (*p* < 0.05) ↑ Restless Sleep 7.1 ± 2.5 cm vs. 3.8 ± 2.3 (*p* < 0.05)
López-Samanes et al. 2021, Portugal [28]	16 males Age: 28 ± 4 yrs	Futsal Tier 2 (Trained)	RCT Double-blind Cross-over	Caffeine 3 mg/kg BM Consumed 17:00	Placebo	Intermittent exercise protocol to simulate futsal match 17:00–19:00	Side Effects Questionnaire	↔ No significant differences in insomnia
Newbury et al. 2022, United Kingdom [29]	5 males 3 females Age: 18 ± 1 yrs	Swimming Tier 3 (Highly Trained)	RCT Double-blind Cross-over	Caffeine 3 mg/kg BM Consumed 16:30	Placebo	Training Session 17:30–20:30	Core Consensus Sleep Diary	↔ No significant differences in subjective sleep measures
Raya-González et al. 2021, Spain [30]	14 males Age: 21 ± 2 yrs	Basketball Tier 4 (Elite)	RCT Double-blind Counter-balanced Cross-over	Caffeine 6 mg/kg BM Consumed 18:30	Placebo	Intermittent exercise protocol to simulate basketball match 19:30–21:00	Side Effects Questionnaire	↑ Insomnia 57% vs. 14% (*p* < 0.05)
Caia et al. 2022, Australia [31]	15 males Age: 23 ± 4 yrs	Rugby League Tier 4 (Elite)	Quasi-experimental	High Salivary Caffeine Post Match	Low Salivary Caffeine Post Match	Ad libitum caffeine consumption prior to and during evening (19:00–21:00) game Salivary caffeine measured 90 min post-game	Actigraphy (Phillip’s Respironics Actiwatch) night prior, night of, night after match Sleep Diary	↔ No significant correlation between post-competition salivary caffeine and sleep parameters
Dunican et al. 2018, Australia [32]	20 males Age: 26 ± 3 yrs	Rugby Union Tier 4 (Elite)	Quasi-experimental	High Salivary Caffeine Post Match	Low Salivary Caffeine Post Match	Ad libitum caffeine consumption prior to and during evening (19:00–21:00) game Salivary caffeine measured before (17:00) and after game (21:30)	Actigraphy (Fatigue Science’s Readiband)	↑ Sleep Latency ↓ Sleep Efficiency

Abbreviations: ↑ increase/higher; ↓ decrease/lower; ↔ no change/difference; BMI, body mass index; KSQ, Karolinska Sleep Questionnaire; mg/kg, milligrams per kilogram; No., number; RCT, randomized controlled trial; REM, rapid eye movement; yrs, years. Athlete calibre has been assigned according to McKay and colleagues participant classification framework [33].

## Data Availability

Data is contained within the article or Supplementary Material.

## Supplementary Materials

The following supporting information can be downloaded at: https://www.mdpi.com/article/10.3390/sports13090317/s1, Table S1: Search Terms; Table S2: Sensitivity analysis [23,24,25,26].
