# Supplementary material for: The Effect of Consuming Caffeine Before Late Afternoon/Evening Training or Competition on Sleep: A Systematic Review with Meta-Analysis
Source: Sports (Basel). 2025 Sep 10;13(9):317. doi: 10.3390/sports13090317 (PMC12473705; doi:10.3390/sports13090317)
Supplement: Supplementary file 1 [file sports-13-00317-s001.zip › Table S1 Search terms.pdf]

Table S1

Summary Table:

Database	Date of Search	Results Retrieved	Limiters Applied
MEDLINE	30 / 9 / 2024	2053	Language: English
CINAHL Plus with Full Text	30 / 9 / 2024	230	Language: English
SPORTDiscus with Full Text	30 / 9 / 2024	639	Language: English

Note: All searches used a combination of controlled vocabulary (e.g., MeSH or CINAHL Headings) and free-text keywords where applicable. Boolean operators (AND, OR) were used to refine results.

Database search results reflect September 2024 queries.

The April 2025 endpoint represents the manuscript submission cutoff date; no formal updated search was conducted as preliminary scanning revealed no newly published eligible studies.

Search Strategy for EBSCOhost Databases

Databases Searched: MEDLINE, CINAHL Plus with Full Text, SPORTDiscus with Full Text

Date of Search: 30 / 9 / 2024

Search Conducted By: Adem Kocak

Search Terms:

(Athlete-related terms)

(Athlete* OR Sport* OR Player* OR Exercise* OR Active OR Elite OR Trained OR Competitive*)

AND

(Caffeine-related terms)

(Caffeine* OR Coffee OR Tea OR "Energy drink*" OR "Pre-workout supplement*" OR Stimulant*)

AND

(Sleep-related terms)

(Sleep* OR Nap OR Naps OR Napping OR Insomnia OR "Sleep quality" OR "Sleep duration" OR "Sleep efficiency" OR "Sleep onset latency" OR "Total sleep time" OR TST OR "Wake after sleep onset" OR WASO OR "Sleep Disturbance*" OR Bedtime*)

Search Filters Applied:

Database	Limiters Applied
MEDLINE	Language: English
CINAHL Plus with Full Text	Language: English
SPORTDiscus with Full Text	Language: English

Online Supplementary Material

Table S1: Full Search Strategies with Controlled Vocabulary Search Terms and Keywords

MEDLINE (via EBSCOhost)

Date of Search: 30 / 9 / 2024

Results Retrieved: 2053

Search Terms:

(Athlete* OR Sport* OR Player* OR Exercise* OR Active OR Elite OR Trained OR Competitive*)

AND (Caffeine* OR Coffee OR Tea OR "Energy drink*" OR "Pre-workout supplement*" OR Stimulant*)

AND (Sleep* OR Nap OR Naps OR Napping OR Insomnia OR "Sleep quality" OR "Sleep duration" OR "Sleep efficiency" OR "Sleep onset latency" OR "Total sleep time" OR TST OR "Wake after sleep onset" OR WASO OR "Sleep Disturbance*" OR Bedtime*)

Filters Applied: Language: English

CINAHL Plus with Full Text (via EBSCOhost)

Date of Search: 30 / 9 / 2024

Results Retrieved: 230

Search Terms:

(Athlete* OR Sport* OR Player* OR Exercise* OR Active OR Elite OR Trained OR Competitive*)

AND (Caffeine* OR Coffee OR Tea OR "Energy drink*" OR "Pre-workout supplement*")

AND (Sleep* OR Nap OR Naps OR Napping OR Insomnia OR "Sleep quality" OR "Sleep duration" OR "Sleep efficiency" OR "Sleep onset latency" OR "Total sleep time" OR TST OR "Wake after sleep onset" OR WASO OR "Sleep Disturbance*")

Filters Applied: Language: English

SPORTDiscus with Full Text (via EBSCOhost)

Date of Search: 30 / 9 / 2024

Results Retrieved: 639

Search Terms:

(Athlete* OR "Elite athlete*" OR "Collegiate athlete*" OR Sport* OR Trained* OR "Physically active*")

AND (Caffeine* OR Coffee* OR Tea* OR "Pre-workout supplement*" OR Stimulant*)

AND (Sleep* OR "Sleep quality" OR "Sleep duration" OR "Sleep disturbance*" OR "Total sleep time" OR Bedtime*)

Filters Applied: Language: English
