# Supplementary material for: The Effect of Consuming Caffeine Before Late Afternoon/Evening Training or Competition on Sleep: A Systematic Review with Meta-Analysis
Source: Sports (Basel). 2025 Sep 10;13(9):317. doi: 10.3390/sports13090317 (PMC12473705; doi:10.3390/sports13090317)
Supplement: Supplementary file 1 [file sports-13-00317-s001.zip › Table S2 (A and B)..pdf]

Table S2: Sensitivity analysis after removal of each study included in the meta-analysis.

(A)

Study Name	Total Mean Difference	95% lower	95% upper	I²	p-Value
Filip-Stachnik, 2022 [28]	-33.81	46.54	114.15	0%	0..21
Miller et al. 2014 [29]	-23.23	7.36	-53.82	11%	0.08
Pontifex et al. 2010 [30]	-41.93	14.63	[-98.49	0%	0.09
Ramos-Campo et al. 2019 [31]	-32.46	38.68	-103.61	0%	0.19

(B)

Study Name	Total Mean Difference	95% lower	95% upper	I²	p-Value
Filip-Stachnik, 2022 [28]	-5.51	1.35	-12.38	0%	0.07
Miller et al. 2014 [29]	-4.40	1.17	-9.98	11%	0.08
Pontifex et al. 2010 [30]	-5.29	1.86	-12.43	0%	0.09
Ramos-Campo et al. 2019 [31]	- 3.03	7.84	- 13.9	0%	0.35
